# Generation of white-eyed *Daphnia magna* mutants lacking *scarlet* function

**DOI:** 10.1371/journal.pone.0205609

**Published:** 2018-11-14

**Authors:** Nur Izzatur Binti Ismail, Yasuhiko Kato, Tomoaki Matsuura, Hajime Watanabe

**Affiliations:** 1 Department of Biotechnology, Graduate School of Engineering, Osaka University, 2–1 Yamadaoka, Suita, Osaka, Japan; 2 Frontier Research Base for Global Young Researchers, Graduate School of Engineering, Osaka University, 2–1 Yamadaoka, Suita, Osaka, Japan; Leibniz Institute on aging - Fritz Lipmann Institute (FLI), GERMANY

## Abstract

The crustacean *Daphnia magna* is an important model in multi-disciplinary scientific fields such as genetics, evolutionary developmental biology, toxicology, and ecology. Recently, the draft genome sequence and transcriptome data became publicly available for this species. Genetic transformation has also been achieved via the introduction of plasmid DNA into the genome. The identification of a screenable marker gene and generation of mutant strains are essential to further advance *D*. *magna* functional genomics. Because crustaceans are closely related to insects, we hypothesized that, similar to *Drosophila* genetic studies, eye color-related genes can function as marker genes in *Daphnia*. We searched orthologs of *Drosophila* eye pigment transporters White, Scarlet, and Brown in the genome of *D*. *magna*. Amino acid sequence alignment and phylogenetic analysis suggested that *D*. *magna* has six *white* and one *scarlet* orthologs, but lacks the *brown* ortholog. Due to the multiplicity of *white* orthologs, we analyzed the function of the *scarlet* ortholog, *DapmaSt*, using RNA interference. *DapmaSt* RNAi embryos showed disappearance of black pigments both in the compound eye and in the ocellus, suggesting that *DapmaSt* is necessary for black pigmentation in *Daphnia* eyes. To disrupt *DapmaSt* using the Crispr/Cas9 system, we co-injected *DapmaSt*-targeting gRNAs with Cas9 mRNAs into eggs and established white-eyed *DapmaSt* mutant lines that lack eye pigments throughout their lifespan. Our results suggest that *DapmaSt* can be used as a transformation marker in *D*. *magna* and the *DapmaSt* mutants would be an important resource for genetic transformation of this species in the future.

## Introduction

The branchiopod crustacean, *Daphnia magna*, commonly referred to as the water flea, is a model organism for various scientific fields such as genetics, evolutionary developmental biology, toxicology, and ecology. Its draft genome sequence, together with transcriptome data, is publicly available [1]. Genetic manipulation tools, such as RNA interference [2] and genome editing [3], have been developed. Because *D*. *magna* is closely related to insect species in arthropods [4], it is suitable for tracing deeper evolutionary roots of developmental programs that have led to tremendous diversity among arthropod species. To investigate its body plan and sex determination, transformations were performed using random integration [5], TALEN-, and CRISPR/Cas-mediated knock-in of plasmid DNA [6,7]. *Daphnia* has also been used as a model in ecotoxicology because it is highly sensitive to environmental changes and artificial chemicals [8]. To evaluate chemicals with hormone-like activities towards *Daphnia* at the level of gene expression, generation of the biosensor daphniid harboring a reporter gene that responds to ecdysteroids or juvenile hormones has been attempted [9,10]. In these transformation experiments, fluorescent protein genes were used as visible markers.

Eye color-related genes function as transformation markers in *Drosophila melanogaster* [11]. The fly has three eye pigment transporters: White, Scarlet and Brown, all of which belong to the ATP-binding cassette (ABC) transporter subfamily G (ABCG) harboring one nucleotide-binding domain (NBD) and one transmembrane domain (TMD) [12,13]. Each “half ABC transporter” is localized in pigment granule membranes of eye pigment cells [14] and forms a heterodimer with one of the other two half ABCGs to create a functional transporter. The White and Scarlet complex transports a tryptophan-derived precursor, 3-hydroxykynurenine, from the cytosol to a pigment granule, resulting in generation of a brown-colored ommochrome pigment, whereas the White and Brown heterodimer transports a guanine-derived precursor that leads to production of a bright red pigment drosopterin [15]. Disruption of *white* impairs transport of both pigments and changes the compound eye color from red-brown to white. Co-integration of the wild-type *white* with gene-of-interest in its mutant allows us to identify transgene integration events [11]. Because eye pigmentation does not require exogenous substrates and is detectable without special equipment such as a fluorescent microscope, eye color genes would be simpler and more convenient to use as transformation markers than fluorescent protein genes. However, generation of a mutant line is required.

*Daphnia* possesses two different eye types, a single bilaterally symmetrical compound eye and a single eye or an ocellus at juvenile and adult stages [16,17]. During embryogenesis under laboratory culture at 22°C, two lateral groups of red ommatidia are first developed at around 36 hours post-ovulation (hpo), gradually coming closer together while turning the color black at 42 hpo, and finally fusing along the midline at 48 hpo. Development of an ocellus is also observed at 36 hpo and this eye appears as black shortly after its emergence. The structure of ommatidia has been shown to be similar between crustaceans and insects [18]. The presence of *white* and *scarlet* orthologs in the genome of closely related daphniid *Daphnia pulex* has been reported [19]. Taken together, it is reasonable to hypothesize that the ABCG orthologs are involved in pigmentation of *Daphnia* eyes. In this study, we annotated orthologs of *white* and *scarlet* in *D*. *magna*. Knockdown of *scarlet* altered the coloration of the compound eye from black to white. We generated a white-eyed *D*. *magna* mutant lacking *scarlet* function by using the CRISPR/Cas9 system, which will be used in the development of a transformation marker system in *D*. *magna*.

## Materials and methods

### *Daphnia* strain and culture conditions

The *D*. *magna* strain (NIES clone) was obtained from the National Institute of Environmental Studies (NIES; Tsukuba, Japan) and has been cultured under laboratory conditions for many generations. The strain was maintained under the following conditions: 80 neonates (under 24 h) were transferred to 5 L of ADaM medium [20] and cultured at 22–24°C under a light/dark photoperiod of 16 h/8 h, respectively. The culture medium was changed after the first week of cultivation. Daphniids were fed once a day with 5.6 × 10^8^ *Chlorella* cells/mL during the first week; after they matured, their offspring were removed once per day and fed with 1.12 × 10^9^ *Chlorella* cells/mL daily.

### Bioinformatics analysis of eye pigment transporters

The genomic locations of each of the orthologs of eye pigment transporters in insects, *scarlet*, *white* and *brown*, were investigated by tblastn searches using the EvidentialGene database: *Daphnia magna* Genome (http://arthropods.eugenes.org/EvidentialGene/daphnia/daphnia_magna_new/BLAST/). Amino acid sequences of orthologs from *Daphnia pulex* and *Drosophila melanogaster* (S1 Table) were obtained from the NCBI database (http://www.ncbi.nlm.nih.gov/) and used as a query. For further confirmation, alignment and phylogenetic trees were constructed using the amino acid sequence of each protein. Insect White, Scarlet, and Brown amino acid sequences were obtained from the database (S1 Table). A multiple alignment was constructed using Clustal W with the following settings: pairwise alignment parameters: gap opening penalty 6.00, gap extension penalty 0.21, identity protein weight matrix; multiple alignment parameters: gap opening penalty 10.00, gap extension penalty 0.24, delay divergent cutoff 30%, gap separation distance 4. The phylogenetic tree was then constructed using the p-distance algorithm and the neighbor-joining method implemented in MEGA version 7 [21]. The phylogenetic tree was rooted to the *Homo sapiens* ABCG2 family.

### RNA interference of *scarlet*

An siRNA targeting the *scarlet* gene was designed and injected into *Daphnia* eggs according to the method established previously [2]. The sequences of siRNAs were as follows: Scarlet, 5ʹ-GGGUCGCAUUGCUUAUCAA-3ʹ; Control, 5ʹ-GGUUUAAGCCGCCUCACAU-3ʹ [22]. Two nucleotides dTdT were added to each 3ʹ end of the siRNA strand. Briefly, eggs were collected from multiple clutches of daphniids within 2–3 weeks of age, just after ovulation, and placed in ice-chilled M4 medium [23] containing 80 mM sucrose (M4-sucrose) to prevent embryonic development, which allowed for injection into one-cell stage of embryos. The siRNA (100 μM) was mixed with 1 mM Lucifer yellow, which was used as a marker to check injection volume. Each injected egg was then transferred into a well of a 96-well plate filled with 100 μl of M4-sucrose. Injected eggs were allowed to develop and each injected individual was screened based on eye pigmentation.

#### Quantitative real-time PCR

The RNAi embryos were collected at 52 h after injection and homogenized with the beads using the Micro Smash machine MS-100 (TOMY; Tokyo, Japan) in the presence of the Sepasol-RNA I reagent (Nacalai Tesque Inc.; Kyoto, Japan). Total RNA was isolated according to the manufacturer’s protocol and followed by phenol-chloroform extraction. The first strand cDNA was synthesized with the Superscript III Reverse Transcriptase (Invitrogen; Carlsbad, CA, USA) using random primers (Invitrogen) according to the manufacturer’s protocol. Quantitative PCR was performed using an Mx3005P real time (RT)-PCR System (Agilent Technologies; CA, USA) with SYBR GreenER qPCR Supermix Universal Kit (Invitrogen) in the presence of a set of primers (*st*-forward 5ʹ-TCTGCGATGAACCAACTACCG-3ʹ and *st*-reverse 5ʹ-TTTCCGACGAAGGCTGATG-3ʹ). The PCR amplifications were performed in triplicate using the following conditions: 2 min at 50°C and 10 min at 95°C, followed by 40 cycles of 15 s at 95°C and 1 min at 60°C. Gel electrophoresis and melting curve analyses were performed to confirm the correct amplicon size and the absence of the nonspecific band. The target mRNA transcript level was normalized to the transcript level of ribosomal protein L32 [24]. Three biological replicates were used in this experiment.

### Cloning of the *D*. *magna scarlet* cDNA

Total RNAs were extracted from embryos at 24–54 h after ovulation and cDNAs were synthesized as described above. Based on the cDNA sequence annotated in this study, gene-specific primers were designed and the partial cDNA fragments were amplified by PCR. The full-length *scarlet* cDNA was determined by 5’ and 3’ rapid amplification of cDNA ends (RACE) with a GeneRacer Kit (Invitrogen) and a SMARTer RACE cDNA Amplification Kit (Clontech Laboratories, Inc.; Mountain View, USA). The oligonucleotides sequences for RACE are shown in S2 Table.

### Knockout of *Scarlet* by CRISPR/Cas9 system

Syntheses of gRNAs and Cas9 mRNAs were performed as described previously [3]. The target site of the *scarlet*-targeting gRNA was 5ʹ-GGTTCACTCGTCGCCTTAATggg-3ʹ (protospacer adjacent motif shown in lowercase). To generate the gRNA expression vectors, the plasmid pDR274 (Addgene plasmid 42250, [25] was digested with *Bsa*I (NEW ENGLAND Biolabs, Connecticut, USA), followed by dephosphorylation with Antartic Phosphatase (NEW ENGLAND Biolabs, Connecticut, USA). A pair of *scarlet-*targeting oligonucleotides were then annealed and ligated into the linearized pDR274 vector using a ligation mix (TaKaRa Bio, Shiga, Japan). To synthesize gRNAs *in vitro*, gRNA synthesis vectors were digested by *Dra*I (NEW ENGLAND Biolabs, Connecticut, USA) and purified by phenol/chloroform extraction. *Dra*I-digested DNA fragments were used as templates for *in vitro* transcription with mMessage mMachine T7 Kit (Life Technologies, California, USA), followed by column purification with miniQuick Spin RNA columns (Roche diagnostics GmbH, Mannheim, Germany), phenol/chloroform extraction, ethanol precipitation, and dissolution in DNase/RNase-free water (Life Technologies, California, USA).

For synthesis of Cas9 mRNA, a template with the T7 promoter was amplified by PCR from pCS-Dmavas-Cas9 [3]. The amplified PCR fragment was used as a template for *in vitro* transcription with the mMessage mMachine T7 Kit. Poly(A) tails were attached to the capped Cas9 RNAs using the Poly(A) Tailing Kit (Life Technologies, California, USA). The synthesized RNA was then column purified using the miniQuick Spin RNA columns, followed by phenol/chloroform extraction, ethanol precipitation, and lastly, dissolution in DNase/Rnase-free water.

gRNA (50 ng/μL) was co-injected with 500 ng/μL Cas9 mRNA into *D*. *magna* eggs as mentioned above. The eye phenotypes of G1 offspring produced by injected G0 mothers were observed under a stereomicroscope. To investigate the Cas9-induced mutations, white-eyed G1 offspring were homogenized in 90 μL of 50 mM NaOH with zirconia beads. The lysate was heated at 95°C for 10 min and then neutralized with 10 μL of 1 mM Tris-HCl (pH 7.5). This crude DNA extract was centrifuged at 12,000 rpm for 5 min and then used as a template in genomic PCR. The gRNA-targeted genomic regions in *st* locus were amplified by PCR with Ex Taq Hot Start Version (Takara Bio, Shiga, Japan). We used the following primers: *st*-fwd-gDNA 5ʹ- GGTCCCCTTCAAACGAGTC -3ʹ and *st*-rev-gDNA 5ʹ-GGACATCTGCAAGCCAA-3ʹ. The PCR products were analyzed by polyacrylamide gel electrophoresis and DNA sequencing.

### Reproduction test

Viability and reproduction were assessed with *scarlet* mutants (MT1 and MT2) and wild-type (WT). Twenty-four neonates (< 24 h old) from each mutant or WT were cultured individually in ADaM medium until they produced the third clutch (approximately 21 days at 22–24°C. The neonates were fed with 5 × 10^6^ cells/mL for 1 week, and thereafter with 1 × 10^7^ cells/mL. The medium was changed and the cumulative number of offspring was counted at each clutch. To measure body length between the top of the compound eye and base of the tail spine [26], ten offspring were randomly chosen at the first instar juvenile stage from the first clutch of each mutant or WT.

## Results

### Identification of *white* and *scarlet* orthologs in *D*. *magna* genome

**Fig 1:**
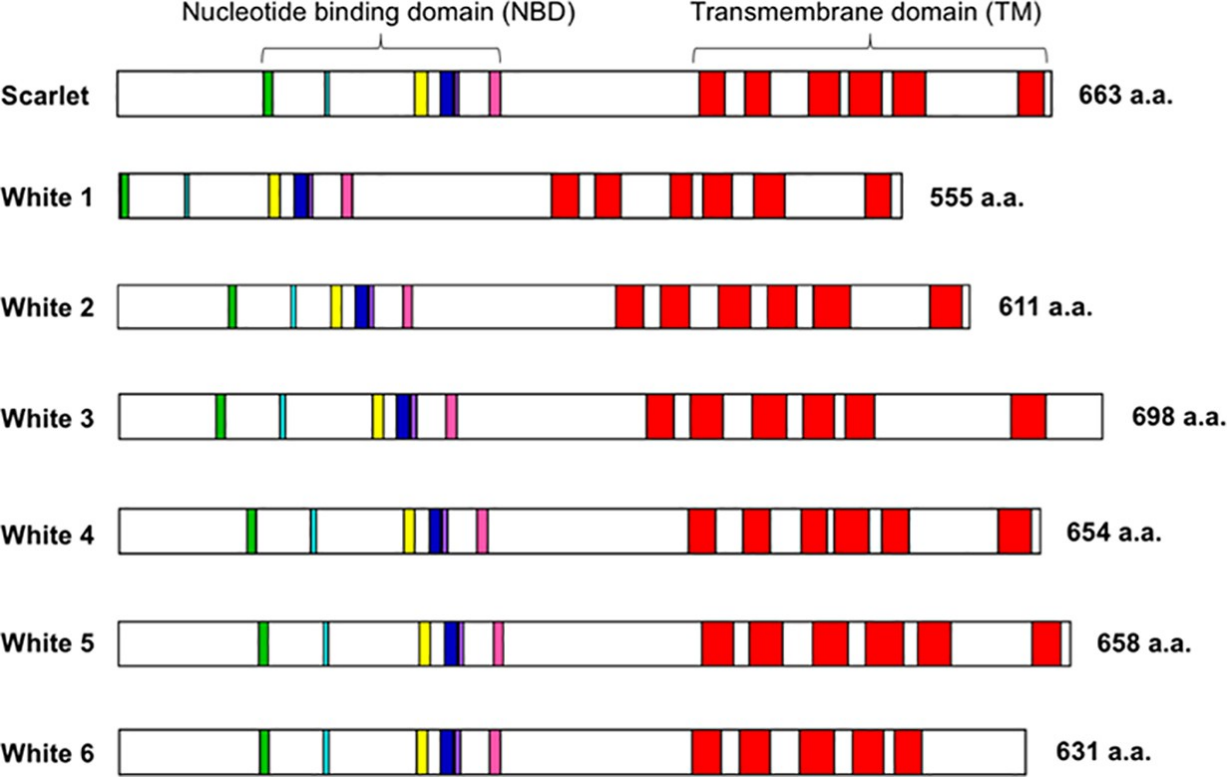
Putative structures of orthologs of eye transporter proteins in *Daphnia magna*. The green, light blue, yellow, dark blue, purple and pink boxes indicate the Walker A/P-loop, Q-loop, ABC signature motif, Walker B, D-loop, and Switch/H-loop. Red boxes show transmembrane regions.

**Fig 2:**
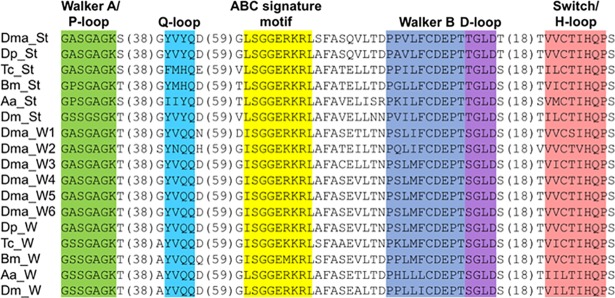
Amino acid alignment of nucleotide binding domains of Scarlet (St), White (W) in *Daphnia magna* (Dma), *Daphnia pulex* (Dp), *Tribolium castaneum* (Tc), *Bombyx mori* (Bm), *Aedes aegypti* (Aa), and *Drosophila melanogaster* (Dm). The accession number of each protein is shown in S1 Table.

**Fig 3:**
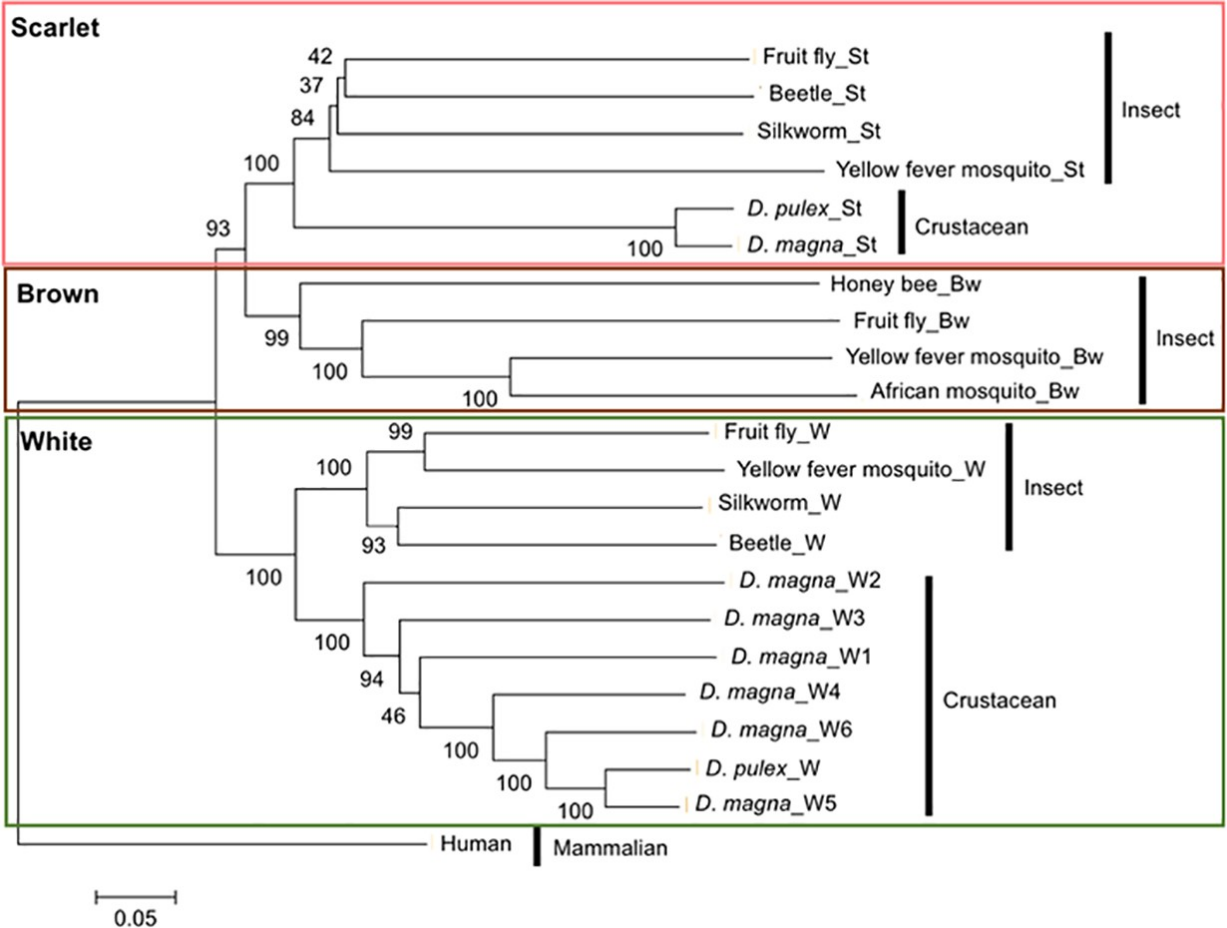
Phylogenetic tree of the amino acid sequences of eye pigment transporters. The percentages of the replicate tree in which the associated taxa clustered together in the bootstrap test (1,000 replicates) are shown next to the branches. The bar indicates branch length and corresponds to the mean number of differences (*P <* 0.05) per residue along each branch. Evolutionary distances were computed using the p-distance method. The accession number of each protein is shown in S1 Table.

To examine whether orthologs of *white* and *scarlet* exist in *D*. *magna*, we searched the *D*. *magna* genome database [1]. We found one *scarlet* and six *white* genes showing high similarity to *D*. *pulex* (Table 1). All of the genes encode one NBD and one TMD at N- and C-terminal regions (Fig 1). We performed alignment of the seven ABC transporters and found sequence conservation of a Walker A motif (or P-loop), Walker B motif, D-loop, Q-loop, and H-motif (or Switch region) in the catalytic core domain, in addition to an ABC signature motif in the α-helical domain (Fig 2,S1 Fig). The phylogenetic analysis showed that one transporter was grouped with the *scarlet* gene and the others with the *white* gene (Fig 3), whereas no brown ortholog was reported in the *D*. *magna* genome. These results suggest that *D*. *magna* has a single *scarlet* ortholog and six *white* orthologs.

**Table 1:** Annotation of *white* and *scarlet* orthologs in *D*. *magna*.

Genes	Genes ID	Scaffold	Location
*scarlet* (*st*)	Dapma7bEVm030711t1	03373	6169–9738
*white1* (*w1*)	Dapma7bEVm011133t1	00575	1594–4046
*white2* (*w2*)	Dapma7bEVm003126t10	03025	416115–419819
*white3* (*w3*)	Dapma7bEVm000188t1	03373	90049–93027
*white4* (*w4*)	Dapma7bEVm028422t1	02019	59050–62778
*white5* (*w5*)	Dapma7bEVm009958t6	02019	63120–66517
*white6* (*w6*)	Dapma7bEVm028439t1	02019	66947–70580

### *scarlet* is necessary for black pigmentation in eyes

**Fig 4:**
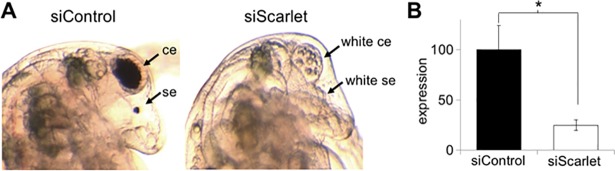
RNA interference of the *scarlet* ortholog in *D*. *magna*. (A) The typical phenotype of embryos injected with the *Scarlet*-targeting siRNA. The cephalic regions of control siRNA-injected (siControl) and scarlet siRNA-injected (siScarlet) embryos are magnified. The ce and se refer to the compound eye and single eye (ocellus). (B) Gene expression profile of *scarlet* in embryos injected with Scarlet siRNAs. Error bars indicate the standard error of the mean (*n* = 3). *P < 0.05 (Student’s t-test).

To find a gene involved in eye pigmentation, we focused on the *D*. *magna scarlet* ortholog (referred as *DapmaSt* hereafter) because multiple *white* genes may lead to gene redundancy that prevents a clear mutant phenotype. To analyze whether *DapmaSt* is required for black eye pigmentation, we reduced its expression by RNA interference (RNAi). We designed the *DapmaSt*-targeting siRNA in a region that is located downstream of the Switch motif (S1 Fig) and is not conserved between DapmaSt and White protein (S2 Fig). We injected it into 29 eggs and evaluated phenotype of the RNAi at 48 h when a wild-type embryo shows black pigment both in a compound eye and in an ocellus (Fig 4A). All of the injected embryos survived and did not have any pigments in both types of eyes (Fig 4A) while 13 embryos injected with the control siRNA showed normal eye pigmentation throughout development. The quantity of *DapmaSt* transcripts was decreased by 75% (Fig 4B). These results suggest that *DapmaSt* is necessary for *D*. *magna* black eye pigmentation.

### Generation of white-eyed *Daphnia* mutants lacking *DapmaSt* gene function

**Fig 5:**
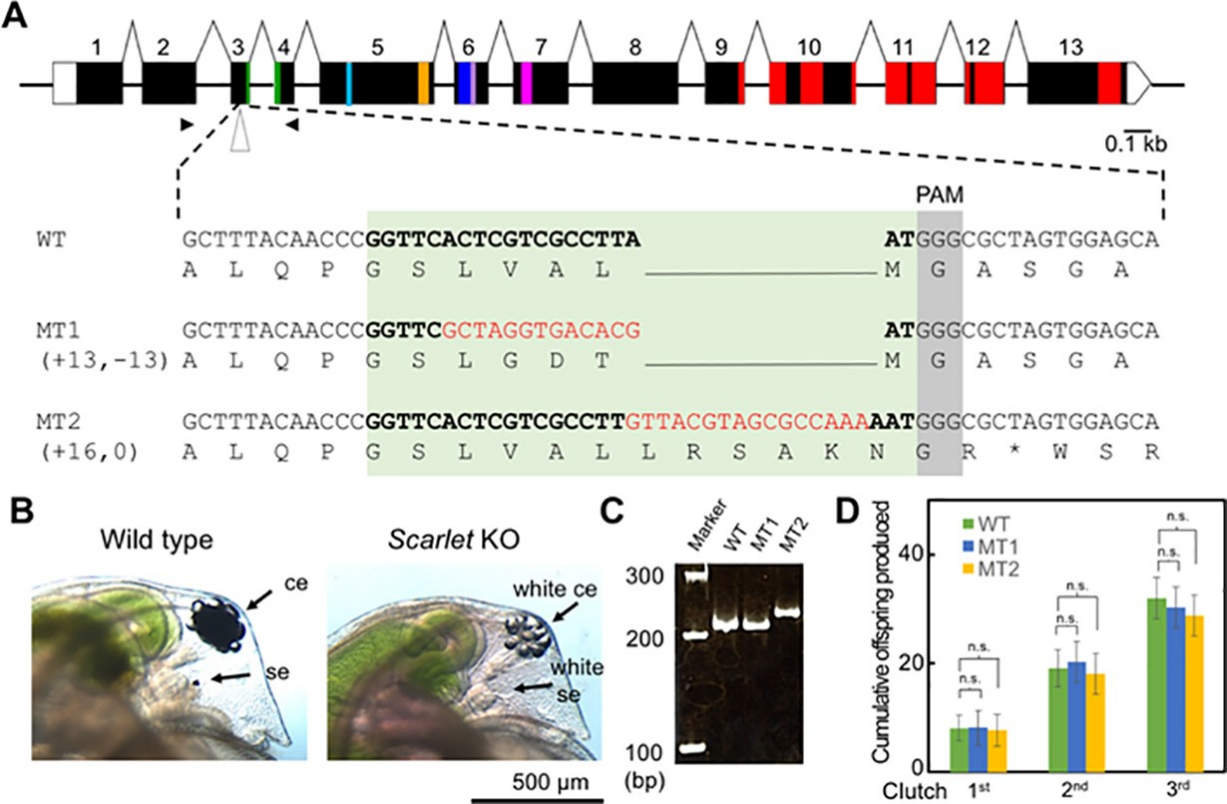
Knockout of the *DapmaSt* in *D*. *magna*. (A) Schematic gene structure of *DapmaSt* and the partial sequences of a wild-type (WT) and two *DapmaSt* mutants (MT1 and MT2). The green, light blue, yellow, dark blue, purple, and pink boxes indicate the Walker A/P-loop, Q-loop, ABC signature motif, Walker B, D-loop, and Switch/H-loop. Red boxes show transmembrane regions. The target sites for gRNA are indicated by bold letters and insertions are indicated by red letters. The protospacer adjacent motif (PAM) sequence is colored in grey. (B) The phenotype of *DapmaSt* mutant with the cephalic regions of a wild type and the *DapmaSt* mutant (MT2) are magnified. The ce and se refer to the compound eye and single eye (ocellus). (C) Polymerase chain reaction for genotyping of WT, MT1, and MT2. The amplified genomic DNA fragments were resolved by agarose gel electrophoresis. (D) Comparison of fecundity between WT, MT1, and MT2. The cumulative number of offspring was counted at each clutch. Error bars indicate the standard error of the mean (*n* = 3). n.s. indicates *P* > 0.05 (Student’s t-test).

As *DapmaSt* RNAi led to a complete loss of eye pigmentation similar to that of the *Drosophila white* mutant, the *DapmaSt* mutant might be suitable as a transformation marker in *D*. *magna*. This prompted us to knockout the *DapmaSt* gene using the CRISPR/Cas9 system. We determined the full-length of the *DapmaSt* ORF sequence by 5ʹ and 3ʹ RACE PCRs in the wild-type strain in this study. The sequences were assembled into a transcript that encodes 663 amino acids. Mapping of the sequenced *DapmaSt* cDNA to the genomic sequence indicated that the gene is 3 kb in length and contains 13 exons (Fig 5A).

To disrupt the *DapmaSt* gene specifically at the genomic level, we designed the gRNA in a region upstream of the Walker A motif (Fig 5A) where its mismatches to the *white* genes were more than 5 bp with PAM (NGG at 3ʹ end), indicating that off-target to the *white* genes is prevented as reported previously [27,28]. We co-injected 50 ng/μL of gRNA with 500 ng/μL of Cas9 mRNA. Of 118 injected eggs, 59 survived to adulthood. We found two adults that produced G1 progenies with white eyes (founder G0 animals) (Fig 5B) and named progeny from each founder G0 animal as MT1 and MT2 mutants. To investigate the indel mutations at the *DapmaSt* locus, we extracted genome DNA from the mutants, amplified a region around the gRNA-targeted site by PCR, and obtained a single PCR fragment for each mutant (Fig 5C). After cloning and sequencing, only one type of indel mutation for each mutant was identified (Fig 5A). In the MT1 mutant, the indel substituted three amino acids, V74G, A75D, and L76T, indicating that these amino acids are essential for DapmaSt transporter activity. In the MT2, a frameshift mutation occurred and introduced a premature stop codon downstream of the gRNA-target site (Fig 5A).

To examine the viability and reproductive ability of the *DapmaSt* mutants, we cultured 24 neonates from each mutant until they matured and produced the third clutch. All of the neonates from each mutant, as well as those from wild-type, survived throughout the experiment.

We counted the number of offspring in the first, second, and third clutches from each mutant and confirmed that fecundity of the *DapmaSt* mutants is similar to that of wild-type (Fig 5D). All of offspring from the both mutants were healthy when they swam out from the brood chamber. We also measured the size of the offspring from each mutant and confirmed that both mutants had offspring that were similar in size to that of the wild-type (S3 Fig). These results demonstrate that white-eyed *DapmaSt* mutants were established.

## Discussion

Rescue of a visible mutant phenotype, such as loss of eye color, is a powerful tool when screening of transformants. However, in *D*. *magna*, no visible mutant phenotypes that do not affect viability have been reported thus far. In this study, we annotated the insect orthologs of genes that code for eye pigment transporters, White and Scarlet, and analyzed the function of the *scarlet* ortholog, *DapmaSt*, by RNA interference and CRISPR/Cas9-mediated knockout. We found that *DapmaSt* mutants do not have any eye pigments throughout their lifespan and therefore represent a promising tool for transformation.

Our annotation demonstrated that *D*. *magna* has six *white*, one *scarlet*, and no *brown* orthologs in *D*. *magna*. Interestingly, *scarlet* is on the same scaffold as one of the *white* paralogs, but at a distance of almost 100 kb. Three of the *white* paralogs occur sequentially on the same scaffold. The latter three appear to be closely related in their phylogeny. We could not exclude the possibility that an error in genome assembly led to overestimation of the number of *white* orthologs and an unfinished assembly prevented us from identifying the *brown* ortholog. However, based on publicly available RNA-seq data [1], all of the genes annotated in this study are expressed in *D*. *magna*. The related daphniid, *Daphnia pulex*, also has multiple *white* orthologs and one *scarlet* ortholog but lacks the *brown* ortholog [19]. These findings suggest that *white* genes annotated in this study are present and may have function in *D*. *magna*. Functional analyses of these genes must be performed to understand their roles in eye pigmentation and the other physiological processes.

Sequences of *white* orthologs are widely conserved in arthropod genomes. In chelicerates [29] and crustaceans, such as Copepoda and Branchiopoda including *Daphnia pulex* [19,30], multiple *white* orthologs have been identified, but only a single *white* ortholog is present in the insects analyzed thus far [31]. In contrast, the *scarlet* ortholog has been identified only in insects and daphniids, whereas the *brown* ortholog seems to be insect-specific [31]. Chelicerates branch at the base of the arthropods and insects are nested within crustaceans [4]. Among crustaceans, branchiopod crustaceans are more closely related to insects than copepods. Thus, *scarlet* may have arisen before the common ancestor of insects and branchiopod crustaceans, whereas *brown* appeared to evolve in the insect clade. However, with the exception of insects, the role of *white* and *scarlet* orthologs in eye pigmentation have yet to be elucidated.

This study revealed that the *D*. *magna scarlet* ortholog, *DapmaSt*, is involved in pigmentation of the compound eye and ocellus. *DapmaSt* RNAi resulted in the white-eyed phenotype. Introduction of mutations into the *scarlet* locus by the CRISPR/Cas system dissipated any eye pigments throughout the individual’s lifespan. Unexpectedly, genotyping of two *DapmaSt* mutants, MT1 and MT2, revealed that both mutants have homozygous indel mutations. This might occur due to gene conversion resulting in the transfers of a mutation from one allele to another. Alternatively, a large deletion that prevented us from amplifying PCR products was possibly introduced into the target site.

The *scarlet* gene was chosen as a candidate marker of transgenesis because there is only one ortholog found in *D*. *magna* genome. For transgenic screening, the ideal marker gene can be detected without using any special equipment, such as fluorescence microscope, and mutation of this gene should not affect the development. Previously, we attempted to use *eyeless*, a gene involved in development of *D*. *magna* eyes, as a transgenesis marker. However, null mutation of this gene is lethal to *D*. *magna* [3,10], thus preventing the gene from being a suitable marker. On the other hand, a null mutation of the *scarlet* gene did not affect reproductive success of *Daphnia*. This observation is important in deciding the suitability of a particular target marker gene.

This work highlights the use of *D*. *magna* orthologs of insect eye pigment transporters and disruption of the *scarlet* gene for generation of mutants with a visible phenotype. The *scarlet* mutant exhibits a white-eyed phenotype, which is similar to the *Drosophila* white-eye mutant. We anticipate that this mutant will be useful for screening of transformants by rescue of the mutant phenotype.

## Supporting information

S1 Table Insect queries and its respective accession number used for phylogenetic tree. (PDF)

S2 Table Oligonucleotide sequences for 5’ RACE and 3’ RACE. (PDF)

S1 Fig Alignment of orthologs of Scarlet (St), White (W), Brown (Bw) in *Daphnia magna* (Dma), *Daphnia pulex* (Dp), *Tribolium castaneum* (Tc), *Bombyx mori* (Bm), *Aedes aegypti* (Aa), *Anopheles gambiae* (Ag), and *Drosophila melanogaster* (Dm). Amino acid sequences corresponding to *DapmaSt* gRNA- and siRNA targeting regions are colored blue and red. The accession number of each protein is shown in S1 Table.
(PDF)

S2 Fig Comparison of St gRNA- and siRNA-targeting sequences with White (W) ortholog sequences. (A) Amino acid sequence alignment of St and W orthologs. (B) Nucleotide sequence alignment.
(PDF)

S3 Fig Body size of wild-type and both mutants (MT1 and MT2) at 0-day old. The body size was measured from the apex of the head to the base of the tail spine. n.s. indicates *P* > 0.05 (Student’s t-test).
(PDF)

## References

[1] Orsini L, Gilbert D, Podicheti R, Jansen M, Brown JB, Solari OS, et al Daphnia magna transcriptome by RNA-Seq across 12 environmental stressors. Sci Data. 2016;3: 160030 10.1038/sdata.2016.30 27164179 PMC4862326

[2] Kato Y, Shiga Y, Kobayashi K, Tokishita S, Yamagata H, Iguchi T, et al Development of an RNA interference method in the cladoceran crustacean Daphnia magna. Dev Genes Evol. 2011;220: 337–345. 10.1007/s00427-011-0353-9 21327957

[3] Nakanishi T, Kato Y, Matsuura T, Watanabe H. CRISPR/Cas-mediated targeted mutagenesis in Daphnia magna. PLoS One. 2014;9: e98363 10.1371/journal.pone.0098363 24878568 PMC4039500

[4] Schwentner M, Combosch DJ, Pakes Nelson J, Giribet G. A Phylogenomic solution to the origin of insects by resolving crustacean-hexapod relationships. Curr Biol. 2017;27: 1818–1824.e5. 10.1016/j.cub.2017.05.040 28602656

[5] Kato Y, Matsuura T, Watanabe H. Genomic integration and germline transmission of plasmid injected into crustacean Daphnia magna eggs. PLoS One. 2012;7: e45318 10.1371/journal.pone.0045318 23028929 PMC3445449

[6] Nong QD, Mohamad Ishak NS, Matsuura T, Kato Y, Watanabe H. Mapping the expression of the sex determining factor Doublesex1 in Daphnia magna using a knock-in reporter. Sci Rep. 2017;7: 13521 10.1038/s41598-017-13730-4 29097757 PMC5668254

[7] Kumagai H, Nakanishi T, Matsuura T, Kato Y, Watanabe H. CRISPR/Cas-mediated knock-in via non-homologous end-joining in the crustacean Daphnia magna. PLoS One. 2017;12: e0186112 10.1371/journal.pone.0186112 29045453 PMC5646780

[8] Ebert D. Ecology, epidemiology and evolution of parasitism in Daphnia [Internet]. Bethesda (MD): National Library of Medicine (US), National Center for Biotechnology Information 2005 Available from: http://www.ncbi.nlm.nih.gov/entrez/query.fcgi?db=Books

[9] Asada M, Kato Y, Matsuura T, Watanabe H. Visualization of ecdysteroid activity using a reporter gene in the crustacean, Daphnia. Mar Environ Res. 2014;93: 118–122. 10.1016/j.marenvres.2013.11.005 24296240

[10] Nakanishi T, Kato Y, Matsuura T, Watanabe H. TALEN-mediated knock-in via non-homologous end joining in the crustacean Daphnia magna. Sci Rep. 2016;6: 36252 10.1038/srep36252 27819301 PMC5098252

[11] Klemenz R, Weber U, Gehring WJ. The white gene as a marker in a new P-element vector for gene transfer in Drosophila. Nucleic Acids Res. 1987;15: 3947–3959. 3108854 10.1093/nar/15.10.3947 PMC340823

[12] Mount SM. Sequence similarity. Nature. 1987; 325: 487. 10.1038/325487c0 3100960

[13] Schmitz G, Langmann T, Heimerl S. Role of ABCG1 and other ABCG family members in lipid metabolism. J Lipid Res. 2001;42: 1513–1520. 11590207

[14] Mackenzie SM, Howells AJ, Cox GB, Ewart GD. Sub-cellular localisation of the white/scarlet ABC transporter to pigment granule membranes within the compound eye of Drosophila melanogaster. Genetica. 2000;108: 239–252. 11294610 10.1023/a:1004115718597

[15] Ewart GD, Howells AJ. ABC transporters involved in transport of eye pigment precursors in Drosophila melanogaster. Methods Enzymol. 1998;292: 213–224. 9711556 10.1016/s0076-6879(98)92017-1

[16] Güldner FH, Wolff JR. Ultrastructure of the compound eye of Daphnia pulex. Z Zellforsch Mikrosk Anat. 1970;104: 259–274. 4913650

[17] Smirnov NN. Nervous system and sense organs Physiology of the Cladocera. 2nd ed Academic Press; 2017 pp. 187–210.

[18] Harzsch S, Melzer RR, Müller CHG. Mechanisms of eye development and evolution of the arthropod visual system: The lateral eyes of myriapoda are not modified insect ommatidia. Org Divers Evol. 2007;7: 20–32.

[19] Sturm A, Cunningham P, Dean M. The ABC transporter gene family of Daphnia pulex. BMC Genomics. 2009;10: 170 10.1186/1471-2164-10-170 19383151 PMC2680897

[20] Klüttgen B, Dülmer U, Engels M, Ratte HT. ADaM, an artificial freshwater for the culture of zooplankton. Water Res. 1994;28: 743–746.

[21] Kumar S, Stecher G, Tamura K. MEGA7: Molecular evolutionary genetics analysis version 7.0 for bigger datasets. Mol Biol Evol. 2016;33: 1870–1974. 10.1093/molbev/msw054 27004904 PMC8210823

[22] Asada M, Kato Y, Matsuura T, Watanabe H. Early embryonic expression of a putative ecdysteroid-phosphate phosphatase in the water flea, Daphnia magna (Cladocera: Daphniidae). J Insect Sci. 2014;14: 181 10.1093/jisesa/ieu043 25399434 PMC5634057

[23] Elendt BP, Bias WR. Trace nutrient deficiency in Daphnia magna cultured in standard medium for toxicity testing. Effects of the optimization of culture conditions on life history parameters of D. magna. Water Res. 1990;24: 1157–1167.

[24] Kato Y, Kobayashi K, Oda S, Tatarazako N, Watanabe H, Iguchi T. Sequence divergence and expression of a transformer gene in the branchiopod crustacean, Daphnia magna. Genomics. 2010;95: 160–165. 10.1016/j.ygeno.2009.12.005 20060040

[25] Hwang WY, Fu Y, Reyon D, Maeder ML, Tsai SQ, Sander JD, et al Efficient genome editing in zebrafish using a CRISPR-Cas system. Nat Biotechnol. 2013;31: 227–229. 10.1038/nbt.2501 23360964 PMC3686313

[26] Mc Kee D, Ebert D. The effect of temperature on maturation threshold body length in Daphnia magna. Oecologia. 1996;108: 627–630 10.1007/BF00329035 28307794

[27] Jiang W, Bikard D, Cox D, Zhang F, Marraffini LA. RNA-guided editing of bacterial genomes using CRISPR-Cas systems. Nat Biotechnol. 2013;31: 233–239. 10.1038/nbt.2508 23360965 PMC3748948

[28] Fu Y, Foden JA, Khayter C, Maeder ML, Reyon D, Joung JK, et al High-frequency off-target mutagenesis induced by CRISPR-Cas nucleases in human cells. Nat. Biotechnol. 2013;31: 822–826. 10.1038/nbt.2623 23792628 PMC3773023

[29] Dermauw W, Osborne EJ, Clark RM, Grbić M, Tirry L, Van Leeuwen T. A burst of ABC genes in the genome of the polyphagous spider mite Tetranychus urticae. BMC Genomics. 2013;14: 317 10.1186/1471-2164-14-317 23663308 PMC3724490

[30] Jeong CB, Kim BM, Lee JS, Rhee JS. Genome-wide identification of whole ATP-binding cassette (ABC) transporters in the intertidal copepod Tigriopus japonicus. BMC Genomics. 2014;15: 651 10.1186/1471-2164-15-651 25096237 PMC4247197

[31] Dermauw W, Van Leeuwen T. The ABC gene family in arthropods: comparative genomics and role insecticide transport and resistance. Insect Biochem Mol Biol. 2014;45: 89–110. 10.1016/j.ibmb.2013.11.001 24291285

