# Supplementary material for: Generation of white-eyed Daphnia magna mutants lacking scarlet function
Source: PLoS One. 2018 Nov 14;13(11):e0205609. doi: 10.1371/journal.pone.0205609 (PMC6235260; doi:10.1371/journal.pone.0205609)
Supplement: S1 Table — (PDF) [file pone.0205609.s001.pdf]

Names	Organism	Gene name (Definition in NCBI)	Accession number
Scarlet	Water flea (*Daphnia pulex*)	ABC protein, subfamily ABCG [Daphnia pulex]	EFX84423
	Beetle (*Tribolium castaneum*)	Protein scarlet [Tribolium castaneum]	NP_001306193.1
	Silkworm (*Bombyx mori*)	Scarlet [Bombyx mori]	NP_001243922.1
	Yellow fever mosquito (*Aedes aegypti*)	Protein scarlet [Aedes aegypti]	XP_001657117.1
	Fruit fly (*Drosophila melanogaster*)	Scarlet protein (Drosophila melanogaster)	AAA82056.1
White	Water flea (*Daphnia pulex*)	ABC protein, subfamily ABCG [Daphnia pulex]	EFX83517.1
	Beetle (*Tribolium castaneum*)	ABC transmembrane transporter [Tribolium castaneum]	NP_001034521.1
	Yellow fever mosquito (*Aedes aegypti*)	Eye pigment transporter [Aedes aegypti]	AAC04894.1
	Silkworm (*Bombyx mori*)	ATP dependent transmembrane transporter [Bombyx mori]	NP_001037034.1
	Fruit fly (*Drosophila melanogaster*)	White [Drosophila melanogaster]	NP_476787
Brown	Honey bee (*Apis mellifera*)	PREDICTED: protein brown isoform X1 [Apis mellifera]	XP_395665.5
	African mosquito (*Anopheles gambiae*)	AGAP007655-PA [Anopheles gambiae str. PEST]	XP_308215.4
	Yellow fever mosquito (*Aedes aegypti*)	AAEL017188-PA [Aedes aegypti]	EJY57661.1
	Fruit fly (*Drosophila melanogaster*)	Brown protein [Drosophila melanogaster]	AAC37214.1
	Human (*Homo sapiens*)	ATP-binding cassette sub-family G member 2 isoform 1 [Homo sapiens]	NP_004818.2

S1 Table. Insect queries and its respective accession number used for phylogenetic tree.
