# Supplementary material for: Generation of white-eyed Daphnia magna mutants lacking scarlet function
Source: PLoS One. 2018 Nov 14;13(11):e0205609. doi: 10.1371/journal.pone.0205609 (PMC6235260; doi:10.1371/journal.pone.0205609)
Supplement: S2 Table — (PDF) [file pone.0205609.s002.pdf]

Names	First primer (5’->3’)	Nested primer (5’->3’)
st fwd (PCR)	CATCTTTGTTGGTTCGTTGACAG	-
st rev (PCR)	GGTTGGTGGCCTTTCTTTGTAG	-
st (5’RACE)	GCCGAGGCTGGTCTCGAACTGAATG	CACGCCTGGCACTCCAATCACTACA
st (3’RACE)	GAATGGCAATTGGACGCCATACGAG	CCCCTGTCCTCTTCTGCGATGAACC

S2 Table. Oligonucleotide sequences for 5’ RACE and 3’ RACE
