# Supplementary material for: Generation of white-eyed Daphnia magna mutants lacking scarlet function
Source: PLoS One. 2018 Nov 14;13(11):e0205609. doi: 10.1371/journal.pone.0205609 (PMC6235260; doi:10.1371/journal.pone.0205609)
Supplement: S1 Fig — Amino acid sequences corresponding to DapmaSt gRNA- and siRNA targeting regions are colored blue and red. The accession number of each protein is shown in S1 Table. (PDF) [file pone.0205609.s003.pdf]

```
Dma_St    1   ------------------MEPTTPVTTSETSSTEFSSSDFTP-IGRLFQQPNIAEKQAEE
Dp_St     1   -------------------MDHTPSPTSDTSSSEFSSADFTPSIGKLFQQPNLAEKQAEE
Tc_St     1   ---------------------------------------------MALALLHISLQESSG
Bm_St     1   --------------MGKKADTTKRSADNSPEKSES----PQPLQSTSYELLPADLVYIDS
Aa_St     1   ---------------------------------------------------------MDG
Dm_St     1   ----------------MSDSDSKRIDVEAPERVEQHELQVMPVGSTIEVPS------LDS
Dma_W1    1   ------------------------------------------------------------
Dma_W2    1   ------------------------------------------------------------
Dma_W3    1   ------------------------MSEKYTITSDETNLKIPHDNLAYDDETDEVKNGKHG
Dma_W4    1   ------------------------MELN----VWEGEYKAVPTNQLLDTTKNGG------
Dma_W5    1   ------------------------MSL-----EKE---KQR----LLQTSRRSGYSSIQG
Dma_W6    1   ------------------------MDL-----HTE---KLKDGHSLNPTFVNLG---FDE
Dp_W      1   -MSSGYGAKPFSDRTCKKELETSTMSL-----HKE---KQR----LIQTSRRTGYSSIAG
Tc_W      1   --MEN-ETEPLLSGVVS----------QINGNSGDSTSSA----------------TSID
Bm_W      1   MTAGNKKQEPLISTSVDNQRVTYNNSPQDGQTPNDSPQS------------------SVG
Aa_W      1   ---------MTINTDDHYAD----NESKSNITSRRYSTSSYQDVDEGINSSFGSNDKS-T
Dm_W      1   --MGQEDQELLIRGGSKHPS----AEHLNNGDSGAASQS-------CINQGFGQAKNYGT
Am_Bw     1   ------------------------------------------------------------
Aa_Bw     1   ------------------------------------------------------------
Ag_Bw     1   ------------------------------------------------------------
Dm_Bw     1   ------------------------------------------------------------

Dma_St    61  IAFLSLTRPSSHTDRP----------IGATLTWRDLSVYVTAPKAKGNAV----------
Dp_St     61  IAFLSLTRPSSHSDRP----------LGATLTWRDLSVYVTTPKAKGNVA----------
Tc_St     61  IFGES----DQFTRKIRTYSQWSPMEEGVTLAWNDVSVYIQTKKNGKTT-----------
Bm_St     61  GIKTSPVKYDSLYPEVEEVFGVPRSPRPCTLVWRDVTVH--VKLKNGR------------
Aa_St     61  TTRLSESVKSELCTSN---------NDTVTLVWQNLTISSQTRNSCS-------------
Dm_St     61  TPKLSKRNSSERSLPLRSYSKWSPTEQGATLVWRDLCVYTNVGGSGQR------------
Dma_W1    61  ------------------------------------------------------------
Dma_W2    61  MKDAEYEAVQTGDNHDVIIPMTANSSQKITYSWENLTAYVNMP-----------------
Dma_W3    61  MANKETALEFSSQHVKG-----VLTSQPVTYTWENIEAEIDIV-----------------
Dma_W4    61  ----DEVLNLSSSG----------ATQPVTYSWENITVFYETT-----------------
Dma_W5    61  D-SRPTSQKRLSNG-KDDFAVAFTTTQPVTYSWENIEIYLETS-----------------
Dma_W6    61  AGENLKSSRRSSLGEKKDSSFSIKRSQPVTYSWENIEIYLEKQ-----------------
Dp_W      61  AESRSRSNKLHSNG-KDDFTVSFTTSPPATYSWENIEIYLETS-----------------
Tc_W      61  LSTFRVP---TYGTTSHPTSKLVPPDERITYSWTEINAFANV------------SPPKTK
Bm_W      61  EVTVAIPQNRNYGAIGG--------IEKVTYTWADVNAFATE------------SRSRGR
Aa_W      61  LIQVWKPK--SYGAVKEQIPQ----HERLTYTWKEIDVFGET--PGDTKKEPLCSRLCCC
Dm_W      61  LRPPSPPE--DSGSGSGQL------AENLTYAWHNMDIFGAVNQPGSG-WRQLVNRTRGL
Am_Bw     61  -----------------MIITEKMEDAGDMIIWNNLTVTVR-----QKRDFFT-------
Aa_Bw     61  ------------MTANKPNDTSTPTG-TVLLEWKNLTVSVRSSSSGQPTSDTG-------
Ag_Bw     61  ------------------------------------------------------------
Dm_Bw     61  --------------MQESGGSSGQGGPSLCLEWKQLNYYVP----AQEQSNYS-------
```

Walker A/ P-loop

```
Dma_St   121  ---------------PFKRVLNNVRGALQPGSLVALMGASGAGKSTLLNALACRCPPGVV
Dp_St    121  ---------------PFKRVLNNVRGALQPGSLVALMGASGAGKSTLLNALACRCPPGVV
Tc_St    121  ----------------CKRIINGVTGAVKAGSLVALMGASGAGKSTLMSTLAHRITGGAF
Bm_St    121  ----------------LKRLVNNVSGIAKPGTLIALMGPSGAGKTTLMSALAHRSPFGTV
Aa_St    121  ------------------TIVHNVNGSLHSGSLVALMGPSGAGKSTLMGALAHRSSAGIT
Dm_St    121  ----------------MKRIINNSTGAIQPGTLMALMGSSGSGKTTLMSTLAFRQPAGTV
Dma_W1   121  ------------------------------------MGASGAGKTTLLDCLTFRNTGKLK
Dma_W2   121  -SGGCFSSLCKKSPPVQKKILDNVSGVIHQGEFLAIMGASGAGKTTLLNCLTYRNTGKLT
Dma_W3   121  -EGRCRKK-----QTTHKRILDHVTGAVQPGEFLAIMGASGAGKTTLLNCLTFRNTGKLK
Dma_W4   121  -PGNCLTRLCKKSPPIRKKILDNVTGIVRPGEFLAIMGASGAGKTTLLNCLTFRNSGQLK
Dma_W5   121  -QGNCFKR----SPPVQKRILDNVTGCVRPGEFLAIMGASGAGKTTLLNCLTFRNTGKLK
Dma_W6   121  -QGNCFKR----QPPIQKRILDNVTGCVRPGEFLAIMGASGAGKTTLLNCLTFRNTGKLK
Dp_W     121  -QGNCFKR----SAPVQKRILDNVTGCVRPGEFLAIMGASGAGKTTLLNCLTFRNTGKLK
Tc_W     121  FFNLIKRKDSP---VQKKHILKNVFGVAYPGELLAILGSSGAGKTTLLNTLTFHTSSNLT
Bm_W     121  RFWSFWKNSSDRMFQQRKQLLRNVNGAAYPGELLAIMGSSGAGKTTLLNTLTFRTPGGVV
Aa_W     121  FSRQKKD------FNPRKHLLKNVTGMAKSGELLAVMGSSGAGKTTLLNALSFRSPPGVK
Dm_W     121  FCNERHI------PAPRKHLLKNVCGVAYPGELLAVMGSSGAGKTTLLNALAFRSPQGIQ
Am_Bw    121  ---NIYNKFQRREYEETLTILKGVSGYAMTGNLVAIMGSSGAGKTTFLATLAGRIKS---
Aa_Bw    121  ---NHWYGRPGHQQKKELTLLRNASGAVRSDNLVAIMGPSGAGKTTLLAAISMRITGSTT
Ag_Bw    121  ------------------------------------MGASGAGKTTLLAAISMRLV--AE
Dm_Bw    121  ---FWNECRK----KRELRILQDASGHMKTGDLIAILGGSGAGKTTLLAAISQRLRG--N
```

Q-loop

```
Dma_St   181  VD--GEIRVNG-RLIDRSFCDMSGYVYQDDIFVGSLTAREHLLFTARLKMNGNW------
Dp_St    181  VD--GEIRING-RLIDRSFCDMSGYVYQDDIFVGSLTAREHLLFTARLKMNGNW------
Tc_St    181  VE--GDVLING-RPIGNYMKYISGFMHQEDIFIGSLTVSEHMNIMARLKLDRKT------
Bm_St    181  ID--GEIIMNG-RPVCSYVDRESGYMHQDDIFAENLTVIEHLTVMARLRMDRRT------
Aa_St    181  TS--GQIRLNG-KRIGPFMYNVSGIIYQDELLCGELTVGEHMHLMACLKLGPSL------
Dm_St    181  VQ--GDILING-RRIGPFMHRNHGYVYQDDLFLGSVSVLEHLNFMAHLRLDRRV------
Dma_W1   181  IL--GQRHLNGEAVNIETLARLSGYVQQNDLFIPTLTVKEHLKFQALLRMDEIF------
Dma_W2   181  VV--GDRYLNGSVVNPDSLARISSYNQQHDLFVGTL------------------------
Dma_W3   181  IT--GTRFLNGAPVNTDALARISGYVQQDDLFIGTLKVGEILRFQALLRMDKHF------
Dma_W4   181  IS--GERYLNGAKVNTDTLARISGYVQQDDLFISTLTVHEHLRFQALLRMDTHL------
Dma_W5   181  IL--GQRFLNGETVNTDTLARISGYVQQDDLFVPTLTVKEHLQFQALLRMDKYL------
Dma_W6   181  IS--GERFLNGAKVNTDTLARISGYVQQDDLFIPTLTVKEHLEFQALLRMEKHL------
Dp_W     181  IS--GDRYLNGEVVNTDTLARISGYVQQDDLFIPTLKVKEHLQFQALLRMDKHL------
Tc_W     181  VS--GLRCVNGIPVSSKTLASQSAYVQQDDLFIGTLTVKEHLIFQALLRMDRDI------
Bm_W     181  AT--GTRALNGQPATPDALTALSAYVQQQDLFIGTLTVREHLVFQAMVRMDRHI------
Aa_W     181  IAPTSVRALNGIPVNAEQLRARCAYVQQDDLFIPALTTREHLVFHAMLRMGKDV------
Dm_W     181  VSPSGMRLLNGQPVDAKEMQARCAYVQQDDLFIGSLTAREHLIFQAMVRMPRHL------
Am_Bw    181  TTG--SVTINGQIISRTIMSVMSGYLPQFDALPTSLTVEEHLLFSCALKTDISR------
Aa_Bw    181  VHG--KVLINGLYVTRTQMKQLTGFVPQYEIALQTLTVAEHLTFVLCLHTHNYLPSSLRS
Ag_Bw    181  VHG--NVLINGLYVSQTQMKRLSGFVPQFEIAVQSLTVREHLSFVVVFKVHTFS------
Dm_Bw    181  LTG--DVVLNGMAMERHQMTRISSFLPQFEINVKTFTAYEHLYFMSHFKMHRRT------
```

```
                                                                  ABC signature
                                                                      motif
Dma_St   241  ----TPYEQNLRVNELLTELGLMKCQNVVIGVPGV-TKGLSGGERKRLSFASQVLTDPPV
Dp_St    241  ----TPYEQNLRVKELLTELGLIKCQNVVIGEPGV-TKGLSGGERKRLAFASQVLTDPAV
Tc_St    241  ----TQQERNSKIHEILKSLGLTKCLNTRIGINGE-SKVLSGGEKKRLAFATELLTDPPI
Bm_St    241  ----STVARKRRVNQLMRQLSLYESRFTRIGGLDG-HKTLSGGERKRLAFATELLTDPGL
Aa_St    241  ----STHRKHLLINDLLTQTNLLQCYHTQIGQIGV-RKTLSGGERKRLAFAVELISRPKI
Dm_St    241  ----SKEERRLIIKELLERTGLLSAAQTRIGSGDD-KKVLSGGERKRLAFAVELLNNPVI
Dma_W1   241  ----SYDERMGRVNEAIQELGLTKCMDTVIG-----HADISGGERKRLAFASETLTNPSL
Dma_W2   241  -----------------------KCADSLIGEPERAIKGISGGEKKRLAFATEILTNPQL
Dma_W3   241  ----SYEERMERVEEVILELGLTKCRNTLVGNPEKGIKGISGGERKRLAFACELLTNPSL
Dma_W4   241  ----TYEERMTRVEEVINELGLTKCSGSMIGHPERGIKGISGGERKRLAFASEVLTNPSL
Dma_W5   241  ----TYSERMTRVGQVIHELGLTKCENTMIGNPERGIKGISGGERKRLAFASEVLTNPSL
Dma_W6   241  ----NYDERMIRVGEVIHELGLKKCENTVIGNPERGIKGISGGERKRLAFASEVLTNPSL
Dp_W     241  ----TYAERMIRVGQVIHELGLSKCENTVIGNPERGIKGISGGERKRLAFASEVLTNPSL
Tc_W     241  ----SYSQRMARVEEVISDLALSKCQNTPIGILGR-IKGISGGEKKRLSFAAEVLTNPKL
Bm_W     241  ----PYAQRMKRVQEVIQELALSKCQNTVIGIPGR-LKGISGGEMKRLSFASEVLTDPPL
Aa_W     241  ----PKSVKMNRVNEVLQELSLAKCADTIIGAPGR-MKGLSGGERKRLAFASETLTDPHL
Dm_W     241  ----TYRQRVARVDQVIQELSLSKCQHTIIGVPGR-VKGLSGGERKRLAFASEALTDPPL
Am_Bw    241  --VQRKFLS----MKLLMELNLIDCKDVLISN-------LSGGQRKRVSLASEMISRPKI
Aa_Bw    241  IHHELKNVGYVAVLRIVNELGLQGCWGTRIAQ-------LSGGERKKVNLAGELLTEPEI
Ag_Bw    241  ---QLKGVQNHRMNQVIKELQLDKCEDTRISN-------LSGGERKKVNLAGELLTEPDI
Dm_Bw    241  ----TKAEKRQRVADLLLAVGLRDAAHTRIQQ-------LSGGERKRLSLAEELITDPIF

            Walker B D-loop
Dma_St   301  LFCDEPTTGLDTFSAERLVFMLKDLTQRGK------------------------------
Dp_St    301  LFCDEPTTGLDTFSAERLVMMLKDLTQRGK------------------------------
Tc_St    301  LFCDEPTTGLDSYSAQKIVTMMNTMASSGK------------------------------
Bm_St    301  LFCDEPTTGLDSSSALKLVSLLRASAAQGK------------------------------
Aa_St    301  LFCDEPTTGLDSYSAGQVVHMIRRLTRSGT------------------------------
Dm_St    301  LFCDEPTTGLDSYSAQQLVATLYELAQKGT------------------------------
Dma_W1   301  IFCDEPTSGLDSFMAYNIIQVLKTIALTGK------------------------------
Dma_W2   301  IFCDEPTSGLDSYMAQNIIEALKNLASKGK------------------------------
Dma_W3   301  MFCDEPTSGLDSFMAQNIVQALKNLASAGK------------------------------
Dma_W4   301  MFCDEPTSGLDSYMAQNIVQVLKNIASTGK------------------------------
Dma_W5   301  MFCDEPTSGLDSYMAQNIVQVLKNIASTGK------------------------------
Dma_W6   301  MFCDEPTSGLDSYMAQNIVQVLKNIASTGK------------------------------
Dp_W     301  MFCDEPTSGLDSYMAQNIVQVLKNIASTGK------------------------------
Tc_W     301  MFCDEPTSGLDSFMALTVMQVLKEMAMTGK------------------------------
Bm_W     301  MFCDEPTSGLDSFMAQNVIQVLKGLAQKGK------------------------------
Aa_W     301  LLCDEPTSGLDSFMAHSVLQVLKGMALKGK------------------------------
Dm_W     301  LICDEPTSGLDSFTAHSVVQVLKKLSQKGK------------------------------
Am_Bw    301  LFLDEPTTGLDRFSAMQVVNALKIISSEST------------------------------
Aa_Bw    301  LFCDEPTTGLDSFNAASVMKTLQCLCANGR------------------------------
Ag_Bw    301  LFCDEPTTGLDSFSALAVLKTLRKIALKGR------------------------------
Dm_Bw    301  LFCDEPTTGLDSFSAYSVIKTLRHLCTRRRIAKHSLNQVYGEDSFETPSGESSASGSGSK
```

```
                                                                                       Switch/
                                                                                       H-loop
Dma_St   361  -------------------------------------TVVCTIHQPSSETFAMFDRLVLL
Dp_St    361  -------------------------------------TVVCTIHQPSSETFAMFDRLVLL
Tc_St    361  -------------------------------------TILCTIHQPSSDIFAMFSQLILM
Bm_St    361  -------------------------------------TVICTIHQPSSELMAHFDKLVLL
Aa_St    361  -------------------------------------SVMCTIHQPSDELFYMFDSVLLL
Dm_St    361  -------------------------------------TILCTIHQPSSQLFDNFNNVMLL
Dma_W1   361  -------------------------------------TVVCSIHQPSSDVFDLFDRILLM
Dma_W2   361  -------------------------------------VVVCTVHQPSSQVFAMFDHVLLM
Dma_W3   361  -------------------------------------TVICTIHQPSSEVFAMFDRILLM
Dma_W4   361  -------------------------------------TVVCTIHQPSSEVFALFDRILLM
Dma_W5   361  -------------------------------------TVVCTIHQPSSEVFALFDRILLM
Dma_W6   361  -------------------------------------TVVCTIHQPSSEVFALFDRILLM
Dp_W     361  -------------------------------------TVVCTIHQPSSEVFALFDRILLM
Tc_W     361  -------------------------------------TVICTIHQPSSEVYSMFDKLLLM
Bm_W     361  -------------------------------------TVVCTIHQPSSELYAMFDKLLIM
Aa_W     361  -------------------------------------TIILTIHQPSSELYCLFDKILLV
Dm_W     361  -------------------------------------TVILTIHQPSSELFELFDKILLM
Am_Bw    361  --------------------------------------VFCTIHQPGMDIYNIFTHVLLL
Aa_Bw    361  ------------------------------------RAVICTIHDPPSQVFQCFSDVILM
Ag_Bw    361  ------------------------------------KAVICTIHHPTSDAFQCFTDIVLV
Dm_Bw    361  SIEMEVVAESHESLLQTMRELPALGVISNSPNGTHKKAAICSIHQPTSDIFELFTHIILM

Dma_St   421  AEGRIAYQG---------------------------------------------------
Dp_St    421  AEGRIAYQG---------------------------------------------------
Tc_St    421  ADGRIAFIG---------------------------------------------------
Bm_St    421  AEGRIAFAG---------------------------------------------------
Aa_St    421  SNGRTAFMG---------------------------------------------------
Dm_St    421  ADGRVAFTG---------------------------------------------------
Dma_W1   421  AEGRTAFFG---------------------------------------------------
Dma_W2   421  AEGRTAFMG---------------------------------------------------
Dma_W3   421  AEGRTAFLG---------------------------------------------------
Dma_W4   421  AEGRTAFLG---------------------------------------------------
Dma_W5   421  AEGRTAFLG---------------------------------------------------
Dma_W6   421  AEGRTAFLG---------------------------------------------------
Dp_W     421  AEGRTAFLG---------------------------------------------------
Tc_W     421  SEGRTAFLG---------------------------------------------------
Bm_W     421  ADGRVAFLG---------------------------------------------------
Aa_W     421  AEGRVAFLG---------------------------------------------------
Dm_W     421  AEGRVAFLG---------------------------------------------------
Am_Bw    421  SDGKTGYFG---------------------------------------------------
Aa_Bw    421  QDGGTVFY-------------------------------------QGPTADRIDFFN---
Ag_Bw    421  RKEALVGIGFAFLLNAGLELAEKLKITMVLRSMPESYHFLASALEARPDADVTMQLVRSK
Dm_Bw    421  DGGRIVYQG---------------------------------------------------
```

```
Dma_St   481  -------------SSSGALGFFESMGYVCPATYNPADFYVQTLAVIPGLEDTS--RSTVR
Dp_St    481  -------------SSSGALGFFESMGYTCPATYNPADFYVQTLAVIPGLEDTS--RSTVR
Tc_St    481  -------------SAASALDFFQKAGYRCPTSYNPADFFIKTLATTPGFEENS--KQCIK
Bm_St    481  -------------NASAALGFFESLGYHCPLTYNPTDYFIKVLALTPGSEAAS--RHAIK
Aa_St    481  -------------KPHEAIQFFDRLGMIRPGNCATAEHFIKCLSTCR---DASD-RIKPE
Dm_St    481  -------------SPQHALSFFANHGYYCPEAYNPADFLIGVLATDPGYEQASQ-RS-AQ
Dma_W1   481  -------------PVPDCLSFFSSQGLSCPSNYNPADFYIRTLATMAGDEAES--KKKIA
Dma_W2   481  -------------PTNKAIDFFASQGMPCPPNHNPADFFIYALASIPGQEEET--NKKVK
Dma_W3   481  -------------PIDDCLRFFSCHGMPCPANYNPADFYIFSLATVPGKEIES--RSKIK
Dma_W4   481  -------------PVGDALSFFSAQGMPCPPNYNPADYYIHTLATVPGHEVES--KKKIK
Dma_W5   481  -------------PVSDALSFFSSQGLPCPPNYNPADYYIHTLAIVPGQEIES--RKKSK
Dma_W6   481  -------------PASDALSFFSSQGLPCPPNYNPADYYIHTLATVPGQEIES--RKKSK
Dp_W     481  -------------PVGDALSFFSAQGLPCPPNYNPADYYIHTLATIPGQEVES--KKKSR
Tc_W     481  -------------SPEEAETFFRELEAPCPRNYNPADYFIQLLAIVPEKEESS--RQAVN
Bm_W     481  -------------SSDEAFQFFKELGAACPANYNPADHFIQLLAGVPGREEVT--RHTID
Aa_W     481  -------------SPYQASEFFSQLGIPCPPNYNPADFYVQMLAIAPNKEAEC--RDTIK
Dm_W     481  -------------TPSEAVDFFSYVGAQCPTNYNPADFYVQVLAVVPGREIES--RDRIA
Am_Bw    481  -------------SLKDATKFFLSLDYECPVGFDESEYYVKLLSRRNPIMYATN------
Aa_Bw    481  -----------------------SIGKEVPANGNPADFYFQLVSPG-----------ATT
Ag_Bw    481  LMDEYHKRQERSGKSSSAAKDYPVIHFPLPINCNPADHYFKLVCDYSQIDHVENDHHLQQ
Dm_Bw    481  -------------RTEQAAKFFTDLGYQLPLNCNPADFYLKTLADKEGKENAG----AVL

Dma_St   541  AICDRFIVTSTAKQIDLLIQFETS------------LGQEMLDLSTKKGHQPAELHQARW
Dp_St    541  AICDRFIVTSTAKQIDLLIQYETS------------LGQEMLELSTKNGHQPVGLHQARW
Tc_St    541  RICDYFAVSDYNKEVNVVVQYEFH------------MGRAVESKIYKLRT---NFNEMFF
Bm_St    541  SICDRFAVSDVAKELDMEIHLEYH------------LMDNEVEDSRRLRG--DSFRPPHF
Aa_St    541  TICDEYERSDIYHQQKLVISSELL------------LSE--YGYRRPLEME-DSQQRHSW
Dm_St    541  HLCDQFAVSSAAKQRDMLVNLEIH------------MAQ--SGNFPFDTEV-ESFRGVAW
Dma_W1   541  EICDVYESSEASRLVSATA-----------KANSTNSNPKIQESDGNKVNN--SPYKVSW
Dma_W2   541  EICDNFESSDMGKDLLQMAKDNQP---VVASANGGDTISGPSNIQFK-----RSPYKASW
Dma_W3   541  YLCDAYDTSEAAKQVKEIVQ----------KEN---NHSTRQ-NINDSENMKKSPYKANW
Dma_W4   541  EFCDTYDTSDAGKSVQEMV-----------IANRSIKSSRSQELDLEPVKIQRSPYKASW
Dma_W5   541  EICDAYESSQAGQQILEIV-----------KANRSFKLSESQEFQLDEVNVRKSPYKASW
Dma_W6   541  EICDSYESSQAGQQILEIV-----------KANRSLNSTESQEFELAEVKAKKSPYKASW
Dp_W     541  EICDAYVVSTAGQQILEIV-----------KANRSFNLTESQEFQLDDVKVKRSPYKASW
Tc_W     541  LICDKFERSNIGVKIALEAA--------TTER--EGGYHDIWMSGES----FKSPYKASC
Bm_W     541  TVCTAFAKSEIGCRIAAEAENAL-----YNERKIQAGLADAPWAMSSTTRAGRSPYKASW
Aa_W     541  KICDSFAVS----PMAREVMEVA-----NSGKNVEEQYYLQPMEGASRTG-----YRSTW
Dm_W     541  KICDNFAIS----KVAR-DMEQL-----LATKNLEKP-LEQPENGYT--------YKATW
Am_Bw    541  ---PKPEDTGPSELIDKICRAFSRSPLSRIPEIKNTRYFEIEPQRKS-----------GC
Aa_Bw    541  FAASEAEEA---------IQRYEIVRKACRQNIARKCLMTRYHQAKIIQKLANDKHRVCR
Ag_Bw    541  QQALERETDS--------KTRYEIVRKCHMENIGKK-----------------DNHHACW
Dm_Bw    541  RAKYEHETDGLYSGSWLLARSYSGDYLKHVQNFKKIR----------------------W
```

Dma_St	601	MVQFIWLLWRAFVDSYRNPAVHTLRIIQK---IAIALLAGLCFHGVLGTRDQKTIQNIQG
Dp_St	601	TIQFLWLVWRAFVDSYRNPAVHTLRILQK---IAIALLAGLCFHGVLGTRDQKTIQNIQG
Tc_St	601	WQKLYWLTYRWFLDLWRDPTLQATKISEK---IVIGIMIGLCYLGTDFTT-QVGIQNVEG
Bm_St	601	YTKIMWLVYRYLLMIIRDPRVQLVRILQK---LAIALTAGVCFLGTPRLT-QAGVQDVQG
Aa_St	601	FYTLNCLIRRNFLCAHRNPQLQYMKLAQR---LVIAVLVGLCFSSTIDLS-QSGAQAVQG
Dm_St	601	YKRFHVVWLRAIVTLLRDPTIQWLRFIQK---IAMAFIIGACFAGTTEPS-QLGVQAVQG
Dma_W1	601	FAQFKAVLWRSFITVIREPTVFKVKMIQT---IFIAVMVALIFQG-HALQFVS-IRNIQG
Dma_W2	601	STQFSTVLWRSWTTVLREPRVLRMKAVQT---IFVAALLALIYKG-QTITDANDIMNING
Dma_W3	601	FQQFSAVIWRSFLSVVRDPQILVVKASSS---IFIALLIALIYQG-QSMDASS-SLNIQG
Dma_W4	601	FAQLRAVMWRSFLSVRREPAVLKVKAFQT---IFIASLIALIFQG-QTYEFQN-VRNFQG
Dma_W5	601	FAQFRAVFWRSLISVLREPAVLRVKAFQT---IFISMLIALIYQG-QTLQYDN-VRNIQG
Dma_W6	601	FAQFRAVLWRSVISVLREPVVLRVKAFQT---IFISAIIALIYQG-QTLEFDN-VRNIQG
Dp_W	601	FAQFRAVFWRSLLSVLREPAVLRVKAFQT---IFISALIALIYQG-QTLQYDN-VRNIQG
Tc_W	601	WAQFKAVLWRSILAVFKEPLLIKVRLLQT---LIISLVIGAIYFG-QDLNQDG-VMNING
Bm_W	601	CTQFRAVLWRSWLSVTKEPMLIKVRFLQT---IMVSILIGVIYFG-QNLDQDG-VMNING
Aa_W	601	WTQFYYVLWRSWLTVLKDPMLVKVRLLQT---AMVATLIGSIYFG-QRLDQDG-VMNING
Dm_W	601	FMQFRAVLWRSWLSVLKEPLLVKVRLIQT---TMVAILIGLIFLG-QQLTQVG-VMNING
Am_Bw	601	MTQFFWLIWRIWVQNRRTIFDSDGWISWFSYFLSMAVVT-TFYMG-INPRTQEGVQNARG
Aa_Bw	601	AKQLMILLHRTTLDSMRKLR---EYLTVTAIFLFTSVVIASLYYD-VRPVSQTSIQDIRG
Ag_Bw	601	PSQLQLLLRRGVIDSVRNIR---QHVIVTLLFLITSITISALYFH-VTPTSQTAIQDIRG
Dm_Bw	601	IYQVYLLMVPFMTEDLRNIR---SGLIAFGFFMITAVTLSLMYSG-IGGLTQRTVQDVGG

Dma_St	661	ALFILTTENTFPALYGALGIFPMEWPLFLRDARGGLYSPSAYYLSKVVALIPGYVIETFV
Dp_St	661	ALFILTTENTFPALYGALGIFPMEWPLFLRDARGGLYSPSAYYLSKVVALIPGYVVETFV
Tc_St	661	IIFLLVSENTFTPMYSILDEFPQKYPLFLREYNSGLYSSFLYFLSRIMAMLPGLIIEPIL
Bm_St	661	ALFIIIAENTFSPMYSVLHMFPEEFPLFNRELKAGLYSTPVYYTARMIALFPGLLIEPVL
Aa_St	661	IIFLIVSENTFLPMYAVLSVFPESFPLFLRERKANLYGTGQFYIAQIVAMLPFVLLESTT
Dm_St	661	ALFIMISENTYHPMYSVLNLFPQGFPLFMRETRSGLYSTGQYYAANILALLPGMIIEPLI
Dma_W1	661	GFLILVSNATFIHIYGVVSVITNEIPTFLREHRIGMYRTDVYFLSKTLADLPSDFLLPFV
Dma_W2	661	ALFILLTNATFQNVYAVVNVFALEQPIFLRDHFNGMYRTDVYVICKMLADLPFQLLYSFL
Dma_W3	661	VLFLFLTNATFENVFAVINTFSFELPIFLREHFNGMYRTDVYFLSKTFAELAIYIFFPFV
Dma_W4	661	ALFVFLTNMTFQSVFGVINDITLELPVFLREHFNGMYRTDIYFLSKTTADLPVYIVFPFL
Dma_W5	661	ALFIFLTNMTFQNVFGVVNVITSELPIFLREHFNGMYRTDIYFLCKTLADLPVYVVFPFI
Dma_W6	661	ALFIFLTNMTFQNVFGVVNAITGELPIFLREHFNGMYRTDIYFICKSIADLPLFILFPFI
Dp_W	661	ALFIFLTNMTFQNVFGVVNVITSELPIFLREHFNGMYRTDIYFLCKTLADLPVYIVFPFV
Tc_W	661	VLFVFLTNMTFQNVFAVINVFSGELPVFLQEHRNGMYRPSIYFISKTLAESPIFIIIPVT
Bm_W	661	AIFMFLTNMTFQNIFAVINVFCSELPIFIREHHSGMYRADVYFLSKTLAEAPVFATIPLV
Aa_W	661	ALFLFLTNMTFQNVFAVINVFSAELPVFLREKRSRLFRVDTYFLGKTIAEVPLFLAVPFV
Dm_W	661	AIFLFLTNMTFQNVFATINVFTSELPVFMREARSRLYRCDTYFLGKTIAELPLFLTVPLV
Am_Bw	661	ALYMMSSEISFTVAYSVIYEFPGQLLIYLREDG--IYSCGPYYVATFCGLVPKAILKAVL
Aa_Bw	661	ALFLMISELVYTISYGVFYTFPAEMPLIRREVGEKSYTLSMYYLHKVLYSVPRAFLESFL
Ag_Bw	661	ALFLMVCELIYTISYAVFYVFSYEMPLLRREVGEQMYRLSAYYVHKALLTVPKAIFHSYL
Dm_Bw	661	SIFMLSNEMIFTFSYGVTYIFPAALPIIRREVGEGTYSLSAYYVALVLSFVPVAFFKGYV

```
Dma_St    721  FVSIAYWLMGLKPEAGAFLYSCWILMVTCNTAAACGTFFSAACESIAVAISFLIPFDYIL
Dp_St     721  FVSIAYWLMGLKPEAGAFLYSCWVLIVTCNTAAACGTFFSAACESIAVAISFLIPFDYIL
Tc_St     721  FVIIVYWLSGLRATTYAFLMTTLAGILTLNSAAACGIFFSNAFDSVPAAMAYLVPFDYVL
Bm_St     721  FTGVVYWLAGLRYSAYAIGLTIFISILVLNVAIACGSFFSCAFGSMPLAIAYLVPFDYSL
Aa_St     721  FILIVYYLAHLRPTILGLLCTVAACTLVMNVSMACGCFFSTMFSSVPMAMSYLVPFDYIL
Dm_St     721  FVIICYWLTGLRSTFYAFGVTAMCVVLVMNVATACGCFFSTAFNSVPLAMAYLVPLDYIF
Dma_W1    721  FTLITYHAIGLNVLADRFFIACAIMILVTNAVTSFGYFVSCLISSTENAINSVTPLVTPL
Dma_W2    721  FIAIPYYPIGFNPDINRFLITVAIMVIVASVAASFGYFVSCLASSPKISSALSAPLIIPL
Dma_W3    721  AFAIPYYIIGLNPLVERFFIGAGIVILVTNVATSFGYFVSCVASTPQVALAISAPMIIPV
Dma_W4    721  FVAIPYYAIGLNPDVGRFFIACGIVILLANVATSFGYVISCMTSSTRVALAVGPPFIIPL
Dma_W5    721  FVTIPYYAIGLNPEAERFFIACGIVILVANVATSFGYMISCLAGSTQVALAMAAPLIIPL
Dma_W6    721  FVLIPYFAIGLNPAADRFFIACGITILVANVASSFGFMISCLAGTTDVALALAPPLIIPL
Dp_W      721  FVTIPYFIIGLNPGAEQFFIACGIVILVANVATSFGYMISCMAGSTQIALAMAAPLIIPL
Tc_W      721  LTSVCYFMIGLNSHGFRFYIACGIMILVANVAISFGYLISCVSRSVSMALSIGPPLVIPF
Bm_W      721  FTTIAYYMIGLNPDPKRFFIASGLAALVTNVATSFGYLISCASSSVSMAASVGPPIIIPF
Aa_W      721  FTSITYPMIGLKSGATYYLTALLIVVLVANVATSFGYLISCASSSISMALSVGPPVIIPF
Dm_W      721  FTAIAYPMIGLRAGVLHFFNCLALVTLVANVSTSFGYLISCASSSTSMALSVGPPVIIPF
Am_Bw     721  FTTVIYFILISQIDLLNFLFYCLITSTAAICGTAYGLMISIMIENIDIATSIMVPIDMLF
Aa_Bw     721  FIGVAYAFVGFSTDFITYCCMSLVSSGASVLAMAYGYLLSCTTGTMNMAIETSNIIFLAF
Ag_Bw     721  FIGIIYGFVQFSTGFATYVGMAAVCTVASLLGVSYGYLFTCITGSLEMSLEAANLIFLLY
Dm_Bw     721  FLSVIYASIYYTRGFLLYLSMGFLMSLSAVAAVGYGVFLSSLFESDKMASECAAPFDLIF

Dma_St    781  FITGGVLISLSSLPDYVSWTKYLSWFLYTNEALSAVQWQNVTSIRCENFNF---------
Dp_St     781  FITGGVLISLSSLPEYVSWTKYLSWFLYTNEALSAVQWQNVTSIKCDESNF---------
Tc_St     781  MLTSGVFVKLSTLPRVFSWTKYLSWLMYSTESISTVQWNGIKNITCDISDQ---------
Bm_St     781  MMTSGIFIKLSSIPRYVAWIRYLSWLMYSNEAMSIVQWDGVENITCTNSNST--------
Aa_St     781  MITSGIFIRIWTIPTVLRWMPFISWMMFASEAISVAQWDGIDYLDCE-GIP---------
Dm_St     781  MITSGIFIQVNSLPVAFWWTQFLSWMLYANEAMTAAQWSGVQNITCF-QESA--------
Dma_W1    781  MYIGGFYIQNSAVPAYLDWMRYLSWFMYGNEALSINQWVGIQFNDTA-------------
Dma_W2    781  MLFGGFFLNNGSVPIYFQWLRYISWLMYGNSALTITQWQGVSFDSPLCNAN---------
Dma_W3    781  LLFGGFFLQNGSVPVYLDWLRYLSWFMYGNEALSINQWYGVEFNNTDCQYVGYNVTEINE
Dma_W4    781  LLFGGFFLRNGSVPVYFDWLRYVSWFMYANEALSINQWNGISFNDTMSP-----------
Dma_W5    781  LLFGGFFLQNGAVPIYFDWMRYISWFMYGNEALSINQWVGVRFNDTV-------------
Dma_W6    781  LLFGGFFLSNEDVPVYFDWMRYISWFMYGNEALSINQWVGVSFNDTS-------------
Dp_W      781  LLFGGFFLQNGAVPFYFEWMRYISWFMYGNEALSINQWSGVTFNDTI-------------
Tc_W      781  LLFGGFFLNVSSIPIYFKWLSFLSWFRYGNGALMINQWENVTN--IQCPN----------
Bm_W      781  MLFGGFFLNSGSVPPYLSWISYLSWFHYGNEALLINQWAGVET--IACTR----------
Aa_W      781  LIFGGFFLNSASVPSYFEYLSYFSWFRYANEALLINQWSTVQEGDIACTR----------
Dm_W      781  LLFGGFFLNSGSVPVYLKWLSYLSWFRYANEGLLINQWADVEPGEISCTS----------
Am_Bw     781  LLTAGMFYNLRSLPTYLTCFKYFSIFFYLNEALSIIYWSRIDDIDCQVSSDLP-------
Aa_Bw     781  MLLGGLYLNLRAFP----LLKYLSFFFFASEGVSVYYWLPIQSIPCNGTSSRLN------
Ag_Bw     781  NLLGGLYLNVVAFP----VSKYLSFFFFASEGVSIYYWQGVQNITCD--EGRN-------
Dm_Bw     781  LIFGGTYMNVDTVP----GLKYLSLFFYSNEALMYKFWIDIDNIDCPVNEDHP-------
```

```
Dma_St   841  ----------------------------PCLHNGQEVMNHF--SFDFSHFSADITSMLFI
Dp_St    841  ----------------------------PCLHNGQEVMKHF--SFDSSRFSVDITSMLFI
Tc_St    841  --------------------------EIPCLTADTQVLEKY--SFSEDNLSRDLWSMLFL
Bm_St    841  --------------------------GVPCVSTGDEVLMQY--DFTSSNLWLDISALLLL
Aa_St    841  --------------------------DRACLHDGDDVLQQY--SFGRTHLMLDFIALITQ
Dm_St    841  --------------------------DLPCFHTGQDVLDKY--TFNESNVYRNLLAMVGL
Dma_W1   841  ------------------------CPDGVCI--GEHILNDY--DYNPDFYYRDILGLCAL
Dma_W2   841  ----------------------VTIAGQTCT--GEDVLDSL--NFEPTYFYRDIGCLFAL
Dma_W3   841  LVPEGAPDEIVNFVEILTGLYTAYEKNVACS--GEDILEIY--NFKPEYFYRDIACLGGL
Dma_W4   841  ------------------------CPQHICT--EDYILKQF--DFNPNNFYRNIGCLFAL
Dma_W5   841  ------------------------CPNGVCT--GEQILKNF--DFDPNLFYRDIGGLCGL
Dma_W6   841  ------------------------CPNNVCT--GEQILASF--DFNP-------PGM*NT
Dp_W     841  ------------------------CPRGVCT--GEKILENF--DFNPNFFYRDIGGLCGL
Tc_W     841  -------------------------ADLPCPKDGHVILETF--HFSEADFVMDVVMLAVL
Bm_W     841  -------------------------ENFTCPASGQVVLETL--SFSQDDFAMDVVNMILL
Aa_W     841  -------------------------ANVTCPSSGQIILETF--NFKVEDFGFDIACLCML
Dm_W     841  -------------------------SNTTCPSSGKVILETL--NFSAADLPLDYVGLAIL
Am_Bw    841  -----------------------------CLKNGEQVLSEYGFKENNLIWDMSGLLILTI
Aa_Bw    841  -------------------------ETITCLANGQAVLEDAGYATSYEALHLNYLVMAVE
Ag_Bw    841  ---------------------------VTCLRNGEAVLQDYGYGTSLDTVYFNYLVMAAE
Dm_Bw    841  -----------------------------CIKTGVEVLQQGSYRTADYTYWLDCFSLVVV

Dma_St   900  YCIFHFLGLMALVRRSRS-------------------------------
Dp_St    900  YCTFHLLGLMAIVRRSRS-------------------------------
Tc_St    900  CIIFHCLSFICLWLKIRKR------------------------------
Bm_St    900  YITFHLLALLALRYRTRRK------------------------------
Aa_St    900  YFLYHALALLFLHRRASKS------------------------------
Dm_St    900  YFGFHLLGYYCLWRRARKL------------------------------
Dma_W1   900  IVGFRVLAFFALLRKTYGKD-----------------------------
Dma_W2   900  MFGFRFVAYLALLKKTSR-------------------------------
Dma_W3   900  ILLFRLFAFFALLGKTRHLFKRHPRSNSKISCGLKRFLLFSSVYLNRKY
Dma_W4   900  IVGFRIIAFLVLLKKT-------YRKN----------------------
Dma_W5   900  IVGFRFLAFFALLSKT-------YRKN----------------------
Dma_W6   900  SVKT------APASTK---------------------------------
Dp_W     900  IVGFRLVAFFALLSKT-------YRKN----------------------
Tc_W     900  IVGFRLVAFLALLVKTWRFK-----------------------------
Bm_W     900  FVGFRFLAYLALLWRTRRAK-----------------------------
Aa_W     900  IVIFRLGALFCLWLRSRSKE-----------------------------
Dm_W     900  IVSFRVLAYLALRLRARRKE-----------------------------
Am_Bw    900  AMNIIGYFGLRRRRKIQTIL-----------------------------
Aa_Bw    900  IVLVHLVAYMLLRKFVRKAGFY---------------------------
Ag_Bw    900  ILVIHFAAYLCLRRFVRRVGFY---------------------------
Dm_Bw    900  AVIFHIVSFGLVRRYIHRSGYY---------------------------
```

S1 Fig. Alignment of orthologs of Scarlet (St), White (W), Brown (Bw) in Daphnia magna (Dma), Daphnia pulex (Dp), Tribolium castaneum (Tc), Bombyx mori (Bm), Aedes aegypti (Aa), Anopheles gambiae (Ag) and Drosophila melanogaster (Dm). Amino acid sequences corresponding to *DapmaSt* gRNA- and siRNA targeting regions are colored with blue and red. The accession number of each protein is shown in S1 Table.
