# Supplementary material for: Generation of white-eyed Daphnia magna mutants lacking scarlet function
Source: PLoS One. 2018 Nov 14;13(11):e0205609. doi: 10.1371/journal.pone.0205609 (PMC6235260; doi:10.1371/journal.pone.0205609)
Supplement: S2 Fig — (A) Amino acid sequence alignment of St and W orthologs. (B) Nucleotide sequence alignment. (PDF) [file pone.0205609.s004.pdf]

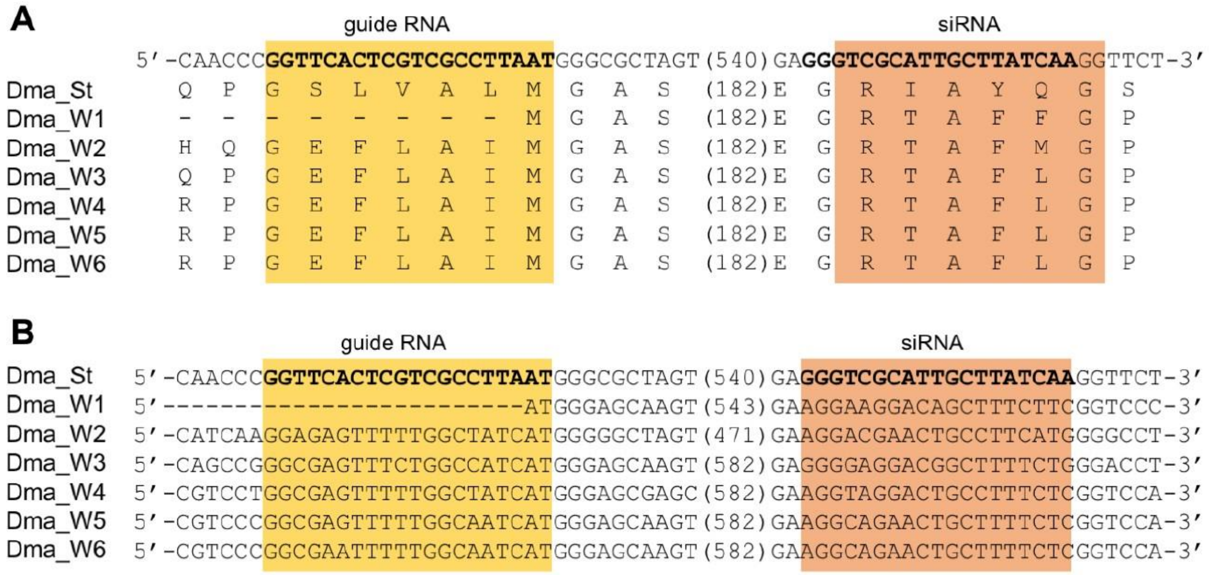

S2 Fig. Comparison of St gRNA- and siRNA-targeting sequences with White (W) ortholog sequences. (A) Amino acid sequence alignment of St and W orthologs. (B) Nucleotide sequence alignment.
