# Supplementary material for: Generation of white-eyed Daphnia magna mutants lacking scarlet function
Source: PLoS One. 2018 Nov 14;13(11):e0205609. doi: 10.1371/journal.pone.0205609 (PMC6235260; doi:10.1371/journal.pone.0205609)
Supplement: S3 Fig — The body size was measured from the apex of the head to the base of the tail spine. n.s. indicates P > 0.05 (Student’s t-test). (PDF) [file pone.0205609.s005.pdf]

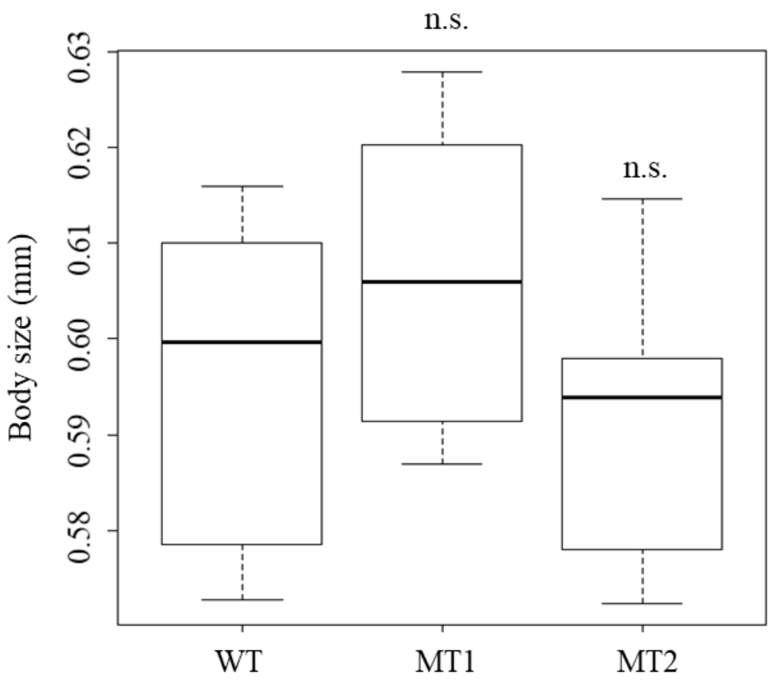

S3 Fig. Body size of wild-type and both mutants (MT1 and MT2) at 0-day old. The body size was measured from the apex of the head to the base of the tail spine. n.s. indicates $P > 0.05$ (Student's t-test)
